# Injury risk assessment using the functional movement screen in college physical education majors: a prospective cohort study

**DOI:** 10.3389/fresc.2026.1777826

**Published:** 2026-03-26

**Authors:** Jie Shao, Xu Fan Bai, Lei Zhu

**Affiliations:** 1 School of Sport Science, Qufu Normal University, Shandong, China; 2 School of Education, Qufu Normal University, Shandong, China

**Keywords:** college students, functional movement screen, injury assessment, physical education, sports injury

## Abstract

**Objective:**

This study aims to explore the application significance of the Functional Movement Screen (FMS) in the scenario of sports injury risk assessment for students majoring in physical education. On the basis of accurately identifying students’ deficiencies in movement function, it further analyzes the corresponding relationship between FMS data and the level of sports injury risk.

**Methods:**

This manuscript is reported in accordance with the STROBE (Strengthening the Reporting of Observational Studies in Epidemiology) statement for cohort studies. The completed STROBE checklist is provided as Supplementary Material (Supplementary File 1). In this prospective cohort study, 355 physical education majors completed a baseline standardized FMS test and were prospectively monitored for sports injuries over one semester. Using GraphPad Prism 9.5.0 software, a Receiver Operating Characteristic (ROC) curve was constructed based on the total FMS scores to realize the assessment of sports injury risk. Meanwhile, a binary logistic regression analysis was adopted to explore the association between the results of the 7 tests included in the FMS and sports injury risk, and the Odds Ratio (OR) was used to quantitatively evaluate the relative impact of relevant factors on sports injury risk.

**Results:**

Data analysis yielded the following findings: (1) The average FMS score of the subjects was 15.623; (2) The ROC curve of the subjects identified a threshold of 14.5 points; (3) The Area Under the Curve (AUC) was 0.8338, indicating diagnostic significance (with a sensitivity of 0.6538, a specificity of 0.8889, and an OR of 9.17). The analysis revealed that when the FMS score was 14.5, the Youden's index reached its maximum value of 0.5427. Since sensitivity is prioritized higher in assessing sports injury risk using total FMS scores, a moderate increase in the number of false positives was deemed acceptable in this study. In addition, as FMS scores are required to be integers, 15 points were finally determined as the optimal cut-off value for judging sports injury risk in this study. Based on the 15-point criterion, there were 160 subjects with a total FMS score ≤15 and 195 subjects with a total FMS score >15. Among them, the risk of sports injury for subjects with a total FMS score <15 was 9 times that of those with a score >15. After adjusting for training volume, previous injury, and sport type, the association remained significant (adjusted OR = 8.21).

**Conclusion:**

A total FMS score of ≤15 was associated with a significantly higher risk of sports injury (OR = 9.17) among physical education majors, suggesting its utility as a screening tool. Students with lower FMS scores have a higher risk of injury during sports compared to those with higher scores.

## 1 Introduction

College students majoring in physical education have a significantly higher risk of sports injuries compared to the general college population due to their high intensity of professional training and frequent participation in extracurricular sports (1, 2). Sports injuries not only directly hinder the development and performance of their motor skills (3), but also often lead to chronic acute injuries and significantly increase the risk of re injury due to untimely treatment or injury training (4, 5). Therefore, establishing effective early screening tools to identify the risk of injury in this population and implement targeted prevention is urgently needed to ensure their training safety and sustainable development in their sports career.

Functional Movement Screen (FMS) is an assessment tool based on the quality of basic movement patterns. Through standardized testing and quantitative scoring, it aims to identify individuals’ functional asymmetry, restrictive or compensatory movements (6, 7). The core idea is that low-quality basic movement patterns may indicate a higher risk of injury. Numerous studies have validated the association between FMS scores and the incidence of sports injuries in different populations, such as elite athletes and military personnel (8–10), confirming its potential as an effective risk screening tool.

However, there are still clear limitations and gaps in the existing evidence when applying FMS to the specific high-risk group of students majoring in physical education in universities. Firstly, most studies have focused on verifying the predictive validity of FMS, but there is a lack of established Cut off Scores with optimal sensitivity and specificity for this population (11, 12). A threshold based on local samples and empirically validated is the key to transforming FMS from a research tool into practical risk management decisions, but currently such data is not sufficient. Secondly, existing research has focused more on the association between FMS total score and injury, while the independent predictive value of seven sub tests (such as squat, hurdle step, shoulder flexibility, etc.) and the specific body part functional defects they reveal lack in-depth analysis of their performance in this population (13, 14). This analysis is crucial for developing personalized corrective training programs (Table 1).

**Table 1:** Basic information of subjects.

Subjects	Age (y)	Height (m)	Weight (kg)	BMI
Male (*n* = 250)	18.97 ± 0.59	1.81 ± 0.05	72.87 ± 6.31	21.97 ± 1.29
Female (*n* = 105）	19 ± 0.8	1.64 ± 0.04	57.2 ± 3.44	21.03 ± 0.38
Total (*n* = 355）	18.97 ± 0.6	1.81 ± 0.05	71.77 ± 6.71	21.91 ± 1.26

Body Mass Index (BMI) is calculated by dividing weight in kilograms by the square of height in meters.

Based on the above gaps, the novelty of this study is mainly reflected in the following three aspects: based on a large sample of prospective data of physical education majors in Chinese universities, through receiver operating characteristic (ROC) curve analysis, an operational FMS injury risk total score threshold applicable to this group is established. Beyond total score analysis, a binary logistic regression model is used to systematically quantify and compare the independent associations between FMS sub item test scores and injury risk, in order to identify the specific action pattern defects that have the most predictive value for this group. A comprehensive evaluation of the practical utility and implementation considerations of FMS in this population includes not only its predictive performance (e.g., AUC and OR), but also inter-rater reliability and interpretability of results.

Therefore, this study aims to achieve the following specific goals by implementing standardized FMS testing and combining it with a semester long prospective injury monitoring: (1) determining the optimal critical value of FMS total score for sports injury risk among students majoring in physical education; (2) Analyze the independent predictive effect of FMS sub item test scores on damage risk; (3) To evaluate the utility of FMS as a screening tool for sports injury risk in this population and provide practical guidance for injury prevention.

## 2 Research objects and methods

This manuscript is reported in accordance with the STROBE (Strengthening the Reporting of Observational Studies in Epidemiology) statement for cohort studies. The completed STROBE checklist is provided as Supplementary Material (Supplementary File 1).

### 2.1 Research objects

This was a prospective cohort study.In this study, students majoring in Physical Education from the School of Physical Education of Qufu Normal University were selected as the test subjects. A total of 364 students were initially recruited, and 9 students who were ineligible or unwilling to participate in the test were excluded, leaving355students as the final subjects. Their physical characteristics are as follows: height - males: 1.81 ± 0.05 meters, females: 1.64 ± 0.04 meters; weight - males: 72.87 ± 6.31 kilograms, females: 57.2 ± 3.44 kilograms; Body Mass Index (BMI) - males: 21.97 ± 1.29, females: 21.03 ± 0.38. After the test staff had fully explained the test content and precautions to the subjects, all subjects signed the informed consent form. This study was approved by the Ethics Committee of Qufu Normal University (No. 2025111) and the Thailand Clinical Trials Registry (TCTR20251119005). Through literature review, it was found that female students could be included in the study if there was no significant difference in FMS scores between males and females.

Inclusion criteria for subjects:(1) Age: ≥ 18 and ≤ 20;(2) Educational background: undergraduate or above;(3) No history of neuromuscular, cardiovascular, pulmonary, vestibular, or rheumatic diseases;(4) No history of cognitive impairment or physical movement disorders;(5) Body Mass Index (BMI): 20 ≤ BMI ≤ 25;(6) No regular medication use.

Exclusion criteria for subjects:(1) Those who experienced severe adverse reactions during or after training and were not suitable for continuing the training;(2) Subjects with poor compliance who failed to follow the experimental procedures, affecting the evaluation of therapeutic effects;(3) Subjects who voluntarily withdrew from the study;(4) Those who were unable or unwilling to receive treatment;(5) Those with acute severe psychological disorders, severe mental illness episodes, or severe cognitive impairment.

To verify the rationality of combining the data of male and female students for analysis, this study conducted the following tests during the data processing stage: firstly, independent sample t-test was used to compare the differences between male and female students in the total score of FMS. The results showed that there was no statistically significant difference in the total FMS score between the two groups (t = 1.12, *p* = 0.264). Secondly, by incorporating gender (as a dummy variable) and its interaction term with the FMS total score into a binary logistic regression model, the moderating effect of gender on the “injury total score” relationship was examined. The results showed that the interaction term between gender and FMS total score had no significant predictive effect on injury risk (*p* > 0.05). Based on the above analysis, merging male and female data for subsequent analysis will not introduce significant bias due to gender differences, and will help improve the statistical power of the overall analysis. Therefore, this study merged and analyzed the data of all 355 subjects.

In additon, To comprehensively characterize the study sample, additional data on sports specialization, previous injury history, and regular training load were collected via a standardized questionnaire. Type of Sport: The participants, all first-year physical education majors, were undergoing general foundational training. Their primary sport backgrounds, based on high-school specialization or current focus, were distributed as follows: Track and Field (*n* = 85, 23.9%), Basketball (*n* = 70, 19.7%), Soccer (*n* = 62, 17.5%), Martial Arts (e.g., Wushu, Taekwondo) (*n* = 48, 13.5%), Gymnastics (*n* = 40, 11.3%), Swimming (*n* = 30, 8.5%), and Others (e.g., Volleyball, Tennis) (*n* = 20, 5.6%). Previous Injury History: Participants were asked if they had sustained a significant musculoskeletal injury (requiring at least one week of training modification or medical consultation) in the past 12 months. A total of 102 participants (28.7%) reported a positive history of such previous injury. (Note: This refers to past injuries, distinct from the prospectively monitored injuries that constitute the study outcome). Training Volume and Intensity: Self-reported average weekly training volume, including mandatory curriculum sessions and voluntary practice, was 14.2 ± 3.1 h. Based on their engagement level, 214 participants (60.3%) identified as training at a “Competitive” intensity (regularly participating in additional team training or preparing for inter-collegiate competitions), while the remaining 141 (39.7%) identified as “Recreational” (meeting curricular requirements only). Table 2 shows Sports Background and Training Characteristics of the Subjects.

**Table 2:** Sports background and training characteristics of the subjects (*n* = 355).

Characteristic	Category/Measure	Value (n, %, or Mean ± SD)
Primary Sport Focus	Track & Field	85 (23.9%)
	Basketball	70 (19.7%)
	Soccer	62 (17.5%)
	Martial Arts	48 (13.5%)
	Gymnastics	40 (11.3%)
	Swimming	30 (8.5%)
	Others	20 (5.6%)
Previous Injury (Past 12 months)	Yes	102 (28.7%)
	No	253 (71.3%)
Weekly Training Volume	Hours (Mean ± SD)	14.2 ± 3.1 h
Self-reported Training Intensity	Competitive Level	214 (60.3%)
	Recreational Level	141 (39.7%)

### 2.2 Methodology

#### 2.2.1 FMS test implementation and reliability

By reviewing relevant literature (15), this study designed and developed a test record form for the Functional Movement Screen (FMS). The basic information of the athletes in this form was filled out independently by the athletes themselves, while the information related to injuries was verified and completed jointly by the instructors and researchers. Prior to the official commencement of the test, the research team provided all participating athletes with a detailed explanation of the complete process, operational standards, and precautions of the FMS test. During the test, the scoring of each FMS movement was conducted collectively by researchers and instructors who had received professional training. In accordance with the unified FMS scoring standards, they graded and recorded each test movement, and finally aggregated and calculated the total FMS score for each athlete. Additionally, a four-month injury monitoring and tracking was carried out. It should be noted that the scorers were not allowed to participate in the rehabilitation training process, so as to prevent potential subjective biases that might arise when the scorers record results due to having prior knowledge of the training situation (Table 3). All FMS scores are independently completed by two professionally trained and certified testers. To ensure consistency in ratings, this study randomly selected 30 participants (approximately 8.5% of the total sample) for inter rater reliability testing. The analysis was conducted using the intra class correlation coefficient (ICC), and the results showed that the ICC range among raters for each item was 0.85–0.92, with a total ICC of 0.90, indicating good consistency in scoring (ICC > 0.75 is considered good reliability). The subsequent analysis is based on the final score agreed upon by the two raters through consultation

1. Test Location: Basketball Gymnasium of the School of Sports Science, Qufu Normal University.
2. Test Time: Basketball classes on February 26, 2025, and March 8, 2025.
3. Test Equipment: Standard FMS™ test kit.

Each athlete was required to complete 7 test movements, with 3 attempts made for each movement. The highest score among the 3 attempts was taken as the final score for that movement. In cases where the quality of movement completion fell between two scores, the lower score was adopted. There were no special requirements for the equipment used by the athletes, as long as it did not hinder the conduct of the test.

**Table 3:** Results and corresponding scores of the 7 FMS test movements.

Score	Score Description
3 points	Capable of completing the functional movement test
2 points	Capable of completing the functional movement test with reduced difficulty, or exhibiting compensatory movements when completing the functional movement test
1 point	Unable to complete the functional movement test
0 points	Experiencing pain during the completion of the functional movement or during the exclusion test

Through a review of relevant domestic literature (16), the following criteria were established for including cases in the statistical analysis: a case must meet either item (1) or item (2) below, and simultaneously satisfy both item (3) and item (4). The specific criteria are as follows:

1. Absence from regular training sessions for a consecutive period of 1 week or more due to sports injuries during training or internal team competitions (including internal round-robin matches, internal teaching matches, etc.);
2. Absence from 2 or more consecutive matches due to sports injuries;
3. The student proactively sought professional assistance from medical support personnel for their own injury conditions;
4. The injury occurred during the training process or competition process.

The injury monitoring period was defined as from March to the early part of July. Sports injuries included muscle injuries, tendon injuries, ligament injuries, joint injuries, bone injuries, and cartilage injuries, while skin abrasions and nerve injuries were not included in the statistical scope.

The FMS test is divided into 4 levels, with a scoring range from 0 to 3 points (3 points being the full score), and the maximum total score is 21 points. For movements that require separate testing of the left and right sides, if the scores of the two sides fall between two values, the lower score is taken as the final score for that test movement.

3points: It indicates that the test movement can be completed smoothly in accordance with the standards, with good symmetry, stability, and flexibility. The movement pattern is free of errors, and there are no compensatory movements or pain.
2points: It means that the movement can be completed, but there are some minor compensatory movements or movement control issues.
1point: Failure to complete the movement.

During the testing of all screening movements, if pain is experienced in the tested joint (including but not limited to such cases), the test score for that screening movement is determined to be 0 points. Meanwhile, in the 3 exclusionary tests, the exclusion results are only categorized as positive or negative: if pain symptoms occur during the test, the result of that exclusionary movement is marked as positive, and the score of the associated screening movement that requires the additional exclusion test is uniformly recorded as 0 points.

#### 2.2.2 Data quality control and handling of ambiguous data

To ensure the robustness of the data, clear protocols were established for handling missing or ambiguous data during both FMS scoring and injury reporting. FMS Scoring: All seven FMS movements were scored independently by two certified raters. In cases of initial disagreement in scores for a movement (e.g., one rater assigned 2 points and the other 1 point), the raters jointly reviewed the video recording of that specific attempt. A consensus score was reached through discussion guided by the standardized FMS criteria manual. If no consensus could be reached or if a video was unavailable, the lower of the two scores was recorded conservatively. No FMS test data were missing for the final 355 participants. Injury Reporting: Injury reports required verification from both the student and their instructor. If a reported injury lacked detail (e.g., unclear mechanism) or if there was a discrepancy between the student's report and the instructor's record, a follow-up interview was conducted with the student by a member of the research team to clarify the circumstances. Injuries that could not be verified as meeting the predefined inclusion criteria (see Section 2.2) after this process were excluded from the “injured group” analysis. The injury monitoring logs were complete for all 355 participants over the study period. Final Dataset: Only participants with complete FMS scores and verified injury status (either confirmed injury or confirmed non-injury for the monitoring period) were included in the final analysis. This rigorous process ensured the internal validity of the dataset used for all statistical analyses.

#### 2.2.3 Injury definition and monitoring protocol

To ensure consistency and reproducibility, a clear operational definition of a reportable sports injury and a structured monitoring process were established prior to data collection. Injury Definition: A reportable sports injury was defined as any musculoskeletal complaint (including muscle, tendon, ligament, joint, bone, and cartilage injuries) sustained during scheduled university training or competition, that resulted in at least one of the following consequences: Time-loss: Inability to complete all or part of a regular training session or match on the day after the injury occurred Medical attention: The student sought evaluation or treatment from certified athletic trainers, physiotherapists, or physicians affiliated with the university's sports medicine program Performance modification: The student could participate but required modification of training volume, intensity, or exercises due to pain or functional limitation for at least 24 h Minor ailments such as skin abrasions, bruises without functional limitation, and illnesses were excluded Injury Monitoring Process: A prospective monitoring approach was employed throughout the defined study period (March to early July). The process involved multiple steps:

Baseline Registration: At the study outset, all participants provided baseline information, including any existing injuries. Weekly Verification: Primary data were collected weekly through a standardized injury report form. This form was completed by the course instructors based on their direct observation and attendance records, capturing any incident leading to time-loss or modified participation. Athlete Self-Report: Participants were instructed to report any new injury or pain that met the above definition immediately to their instructor and to the research team via a designated communication channel (e.g., dedicated email or messaging app), regardless of whether it caused full time-loss. Triangulation and Verification: Any reported injury was cross-verified by at least two sources: the instructor's report, the student's self-report, and, when applicable, medical records from the sports medicine clinic. Discrepancies were resolved through follow-up interviews with the involved student and instructor. Data Recording: For each verified injury, the following details were recorded on a standardized case report form: date of onset, mechanism (e.g., acute traumatic or gradual onset), location, suspected type, and the resulting activity modification (full rest, modified training, or no modification). This structured protocol ensured that injury data were captured systematically, minimizing recall bias and ambiguous reporting.

### 2.3 Data analysis

This study selected different statistical software based on the characteristics of the analysis task to balance analysis efficiency and professional graphical output. Microsoft Excel is used for preliminary data organization and descriptive statistics. GraphPad Prism 9.5.0 software is used to plot Receiver Operating Characteristic (ROC) curves, calculate Area Under the Curve (AUC), and determine the optimal diagnostic threshold. To evaluate the stability and generalization ability of the obtained critical values and diagnostic performance indicators, this study used Bootstrap self-service method for internal validation. The specific process is as follows: 1,000 Bootstrap samples (each sample size *n* = 355) are repeatedly extracted with replacement from the original samples (*n* = 355); Repeat the complete ROC analysis on each Bootstrap sample, recalculate AUC, optimal threshold, and their corresponding sensitivity and specificity; Finally, based on the results of these 1,000 repeated samples, calculate the mean, standard deviation, and 95% confidence interval of AUC, sensitivity, and specificity. SPSS 27.0 software is used to perform independent sample t-test, chi square test, and binary logistic regression analysis. Measurement data is expressed as mean ± standard deviation (x ¯± s). By analyzing FMS scores systematically, determine the threshold of sports injury risk for students majoring in physical education, and explore in depth the relationship between functional movement screening scores and sports injury risk.

To control for the influence of potential confounding factors on the association of “FMS injury”, this study adopted two strategies: firstly, during the inclusion stage of the study subjects, some demographic and health status confounding was controlled through uniform inclusion and exclusion criteria (such as age range, BMI range, and absence of active disease). Secondly, after the main analysis, a sensitivity analysis was conducted to correct for confounding factors. We will include the identified major potential confounding factors [such as weekly training hours, past injury history (yes/no), and major exercise type (categorical variable)] as covariates in the binary logistic regression model. In this model, the injury status (yes/no) is used as the dependent variable, the FMS total score (≤ 15 vs. > 15) is used as the core independent variable, and the above confounding factors are used as covariates. The adjusted odds ratio (aOR) and its 95% confidence interval are calculated to evaluate the independent effect of FMS score on injury risk.

## 3 Results

### 3.1 Comparison of total FMS scores between students with sports injuries and healthy students

**Figure 1:**
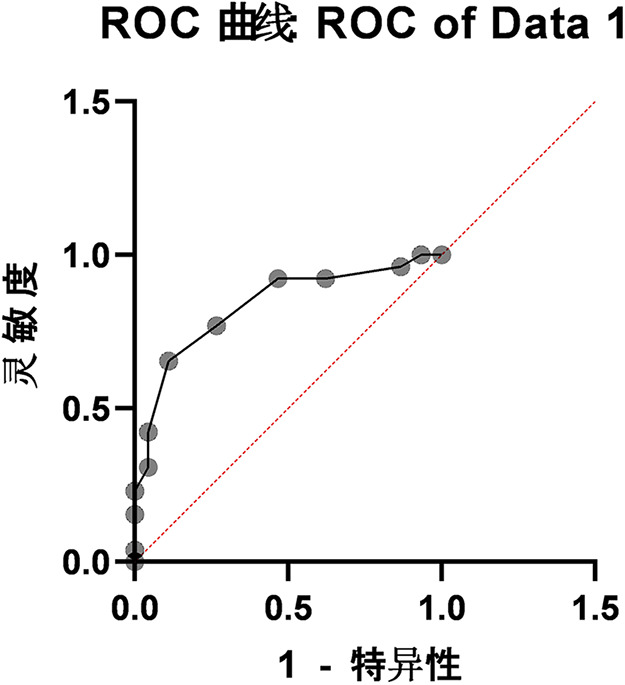
ROC curve chart.

After a semester of follow-up, the FMS scores of the355students were correlated with the actual injury monitoring data. The subjects were divided into an Injured Group and a Healthy Group based on whether they had suffered injuries. The specific data on the total FMS scores of the two groups are presented in Table 4. An independent samples t-test was conducted to statistically analyze the total FMS scores of the two groups, and the results showed that the t-value was 5.723 with a *P*-value < 0.001, indicating a statistically extremely significant difference. The Area Under the Curve (AUC) was 0.8338 (Standard Error: 0.05237, *p* < 0.0001). (See Table 5 and Figure 1). The total FMS score was 13.57 ± 2.59 for the Injured Group and 16.64 ± 1.90 for the Healthy Group.0).

**Table 4:** Total FMS scores of students in the Two groups.

Group	Sample Size (n)	Minimum Score	Maximum Score	Mean Total Score	Standard Deviation
Healthy Group	225	12	20	16.64	1.90
Injured Group	130	8	19	13.57	2.59

**Table 5:** Area under the ROC curve.

Indicator	Original value	mean ± standard deviation	Bootstrap 95% CI
AUC	0.8338	0.835 ± 0.031	0.782–0.902
sensitivity	0.6538	0.648 ± 0.042	0.567–0.725
specificity	0.8889	0.881 ± 0.025	0.834–0.927

### 3.2 FMS score results of subjects

#### 3.2.1 ROC curve analysis

Construct ROC curve based on the total FMS score of the subjects (Figure 1). The original analysis results showed that the area under the curve (AUC) was 0.8338 (standard error: 0.05237, *p* < 0.0001), indicating a good level of discrimination for injury risk prediction, with an asymptotic 95% confidence interval of 0.7311–0.9364 (Table 5). To verify the stability of the model, further report the internal validation results of Bootstrap. After 1,000 rounds of resampling verification, the Bootstrap 95% confidence interval for AUC is 0.782–0.902. The average sensitivity calculated based on Bootstrap samples is 0.648 ± 0.042, and the average specificity is 0.881 ± 0.025. The validation results are highly consistent with the performance indicators of the original samples (sensitivity 0.6538, specificity 0.8889), indicating that the model for predicting damage risk based on FMS total score has good robustness.

#### 3.2.2 Calculation of FMS Score Damage Prediction Threshold

Based on the analysis results of the ROC curve, the Jordan index corresponding to different FMS total scores was calculated, and the results are shown in Table 6.

**Table 6:** Yoden Index of ROC curve.

FMS Score	sensitivity	specificity
< 9.000	0.03846	1.000
< 10.50	0.1538	1.000
< 11.50	0.2308	1.000
< 12.50	0.3077	0.9556
< 13.50	0.4231	0.9556
< 14.50	0.6538	0.8889
< 15.50	0.7692	0.7333
< 16.50	0.9231	0.5333
< 17.50	0.9231	0.3778
< 18.50	0.9615	0.1333
< 19.50	1.000	0.06667

Analysis shows that when the total score of FMS is 14.5, the Yoden index reaches its maximum value (0.5427). Based on this, this study sets 15 points (rounded to 14.5) as the critical value for dividing the risk of sports injuries. Divided by 15 points, there were 160 subjects with FMS total score ≤ 15 points and 195 subjects with FMS total score>15 points. The specific distribution is shown in Table 7.

**Table 7:** Statistics of subjects with FMS scores>15 and ≤ 15.

FMS Score Range	Number of Non-injured Participants	Number of Injured Participants	Total
≤15	60	100	160
>15	165	30	195
Total	225	130	355

To analyze the impact of different FMS total scores (bounded by critical values) and injury status on the distribution of subjects, this study used chi square test for statistical analysis. The results showed that the Pearson chi square value was 16.80 (*p* < 0.001). The calculated odds ratio (OR) is 9.17. Moreover, to control for potential confounding factors, we further included weekly training duration, past injury history (yes/no), and primary exercise as covariates in the binary logistic regression model along with FMS grouping (≤ 15 points vs. >15 points). The adjusted analysis results showed that FMS total score ≤ 15 remained an independent predictor of sports injury risk, with an adjusted odds ratio (OR) of 8.21 (95% CI: 4.05–16.65, *p* < 0.001).

#### 3.2.3 Analysis of scores of each FMS test item and their application in sports injury risk assessment

 Table 8 presents the statistical results of scores for each FMS test item. It can be observed that the scores of the injured group were significantly lower than those of the healthy group. Among all test items, the healthy group achieved notably higher average scores in the Squat and Active Straight-Leg Raise tests compared to the injured group. Additionally, the scores of each test item in this study were slightly higher than those reported in most previous studies (17, 18).

**Table 8:** Scores of each FMS test item.

Group	FMS评分						
	Squat	Hurdle Step	In-Line Lunge	Shoulder Mobility	Active Straight-Leg Raise	Trunk Stability Push-Up	Rotary Stability
Healthy	2.57 ± 0.5	2.08 ± 0.59	2.51 ± 0.55	2.3 ± 0.68	2.8 ± 0.4	2.7 ± 0.51	2.34 ± 0.6
Injured	1.92 ± 0.91	1.8 ± 0.66	2.08 ± 0.63	1.8 ± 0.66	2.39 ± 0.67	2.52 ± 0.71	1.96 ± 0.63

 Table 9 shows the results of binary logistic regression analysis, where injury status was set as the dependent variable (coded as 0 for “no injury” and 1 for “injury”), and the scores of each FMS test item were set as independent variables (coded as natural numbers ranging from 0 to 3). The analysis results indicated that among the independent variables included in each regression model, only the Trunk Stability Push-Up failed to pass the statistical significance test (*P* < 0.05), and the 95% confidence interval of Exp(B) included 1. This suggests that all variables except the Trunk Stability Push-Up had a statistically significant impact on the dependent variable (injury status).

**Table 9:** Variables in the binary logistic regression equation.

Steps	Single item score	B	Std. Error	Wals	df	Significance	Exp(B)	95% Confidence Interval for Exp(B)	
								Upper Bound	Lower Bound
1	Squat Score	−1.462	.481	9.254	1	0.002	0.232	0.090	0.594
	Constant	2.794	1.126	6.159	1	0.013	16.340		
2	In-Line Lunge Score	−1.338	0.355	14.216	1	0.000	0.262	0.131	0.526
	Constant	2.537	0.826	9.431	1	0.002	12.643		
3	Shoulder Mobility Score	−1.073	0.277	15.017	1	0.000	0.342	0.199	0.588
	Constant	1.664	0.586	8.069	1	0.005	5.280		
4	Hurdle Step Score	−0.727	0.298	5.969	1	0.015	0.483	0.270	0.866
	Constant	0.864	0.595	2.107	1	0.147	2.373		
5	Active Straight-Leg Raise Score	−1.376	0.352	15.298	1	0.000	0.253	0.127	0.503
	Constant	3.067	0.942	10.603	1	0.001	21.485		
6	Trunk Stability Push-Up Score	−0.496	0.423	1.372	1	0.242	0.609	0.266	1.396
	Constant	0.756	1.138	0.441	1	0.506	2.130		
7	Rotary Stability Score	−1.010	0.317	10.153	1	0.001	0.364	0.196	0.678
	Constant	1.629	0.698	5.452	1	0.020	5.098		

As shown in the table above, all seven independent variables (scores of each FMS test item) were included in the regression equation, and their regression coefficients (B) were negative. This indicates that lower scores in these FMS test items were associated with a higher likelihood of sports injury.

## 4 Discussion

The Functional Movement Screen (FMS), proposed by sports expert Gray Cook (6), is an assessment tool primarily designed to evaluate the quality of an individual's fundamental movement patterns. This screening method assesses seven basic movement patterns, including deep squat, hurdle step, inline lunge, shoulder mobility, active straight-leg raise, trunk stability push-up, and rotary stability, to identify asymmetries, restrictions, or compensatory movements in an individual's movement performance. Unlike traditional “isolated muscle testing”, FMS focuses on the integrity and continuity of movements, reflecting the functional state of the body during the coordinated work of multiple joints and muscle groups (19). Its core value lies in evaluating an individual's motor ability from a “functional” perspective, rather than merely focusing on isolated muscle strength or joint range of motion, thereby facilitating the detection of potential functional deficiencies.

In this study, FMS tests were conducted on 355 subjects, and independent samples t-test was applied to analyze the test scores. The results showed that the FMS scores of students with injuries were significantly lower than those of healthy students. Therefore, FMS can be used as an effective tool for assessing the risk of sports injuries (20).

### 4.1 Analysis of sports injury risk

#### 4.1.1 Analysis of ROC curve and sports injury

The Receiver Operating Characteristic (ROC) curve is a visualization tool used to evaluate the performance of binary classification models. It is widely applied in medical diagnosis to assess the diagnostic value of detection indicators for diseases. With the false positive rate (FPR) as the *x*-axis and the true positive rate (TPR, also known as sensitivity) as the *y*-axis, the curve is plotted by continuously adjusting the classification threshold, thereby dynamically demonstrating the model's ability to distinguish between positive and negative samples under different thresholds. Specifically, the false positive rate refers to the proportion of samples that are actually negative but incorrectly predicted as positive, while the true positive rate (sensitivity) represents the proportion of samples that are actually positive and correctly identified as positive (21).

The position of the ROC curve intuitively reflects the model's performance. Ideally, the curve should be close to the upper-left corner, indicating that the model can maintain high sensitivity while achieving a low false positive rate. In contrast, a curve approaching the diagonal line from the origin (0,0) to (1,1) suggests that the model's discrimination ability is comparable to random guessing, thus being relatively poor. As a key indicator for measuring ROC curve performance, the Area Under the Curve (AUC) ranges from 0 to 1. An AUC value closer to 1 indicates better classification performance of the model; when AUC equals 1, the model can perfectly distinguish between the two types of samples, and when AUC equals 0.5, the model has no effective discrimination ability (22).

In this study, GraphPad Prism 9.5.0 software was used to generate the ROC curve based on students’ FMS scores. The calculated AUC was 0.8338 (95% CI: 0.7311–0.9364; *p* < 0.0001). According to widely accepted benchmarks for diagnostic accuracy (e.g., an AUC of 0.5 indicates no discrimination, 0.7–0.8 is considered acceptable, 0.8–0.9 is considered excellent, and >0.9 is outstanding), the obtained AUC of 0.8338 indicates excellent discriminatory ability of the FMS total score in identifying students at higher risk of sports injury (22). This strongly supports the practical value of the FMS as a screening tool in this population.

#### 4.1.2 Discussion on FMS risk threshold and clinical decision making

This study determined through ROC analysis that a total FMS score of 14.5 points (rounded to 15 points) is the optimal threshold for distinguishing the risk of sports injuries among students majoring in sports (Yoden index=0.5427). The selection of this integer threshold is based on a trade-off between two aspects: firstly, in the context of injury screening, sensitivity (to avoid missing high-risk individuals) is usually considered to be more prioritized than specificity, so a moderate increase in false positive rate (1-specificity) can be accepted to ensure that more potentially risky individuals are identified (23). Secondly, the FMS score is an integer system, rounded up to 15 points for practical operation and clinical judgment. The positive likelihood ratio (+LR) corresponding to this threshold (15 points) is 5.88, indicating that individuals with positive screening results are 5.88 times more likely to be injured than uninjured, demonstrating good risk assessment efficacy. The odds ratio (OR = 9.17) of this study showed that students with scores below 15 had a risk of injury that was about 9 times higher than those with high scores, which was slightly lower than Kiesel et al.'s report on professional athletes (OR = 11.67) (24). This may be related to the fact that the study subjects were college students and the exercise load and injury types were different.

### 4.2 Analysis of scores of individual FMS test items and sports injury risk

The Functional Movement Screen (FMS) comprises seven individual test items. When assessing an athlete's injury risk, evaluation should not be limited to the total score of these seven items; instead, each item must be analyzed independently. This approach enables the identification of potential issues related to flexibility, stability, and bilateral movement symmetry in specific body regions (25). Li Yongming (26), through research on elite athletes, emphasized that greater attention should be paid to the scores of individual FMS tasks rather than solely relying on the total score.

As indicated by the average scores of each FMS test item in Table 8, the overall performance of the subjects was relatively high. This observation may be attributed to the fact that the study participants were first-year college students majoring in Physical Education, a group characterized by high physical activity levels and superior physical fitness. Among the seven test items, the “Trunk Stability Push-Up” and “Active Straight-Leg Raise” yielded the highest average scores, while the “Hurdle Step” and “Shoulder Mobility” tests resulted in the lowest scores. This disparity is closely linked to the functional characteristics of these specific movements:

The Hurdle Step test involves single-leg stance and dynamic extension of the contralateral limb. The lower scores observed in the injured group may reflect deficiencies in lower limb dynamic stability and hip mobility, which manifest as poor performance during dynamic single-leg movements similar to hurdling.

Shoulder Mobility directly relates to the range of motion of the upper extremities. Reduced scores in the injured group could be indicative of pre-existing shoulder injuries or indirect limitations caused by issues in other regions (e.g., restricted thoracic mobility), which compromise shoulder movement flexibility.

Binary Logistic Regression Analysis is a statistical method used to examine the relationship between independent variables and a binary dependent variable (i.e., a variable with only two possible outcomes). Widely applied in fields such as medicine, it quantifies both the direction and magnitude of the impact of independent variables (which can be either continuous or categorical, with categorical variables requiring coding during analysis) on the binary dependent variable (typically coded as 0 and 1 to represent two distinct outcomes). also employed this method to validate the association between scores of individual FMS test items and sports injury risk (27).

The Binary Logistic Regression Analysis results presented in Table 9 reveal the following key findings:

Scores from six of the seven FMS movement patterns—including Deep Squat, Hurdle Step, Inline Lunge, Shoulder Mobility, Active Straight-Leg Raise, and Rotary Stability—exhibited statistically significant associations with sports injury risk, indicating their utility as valid predictors of such risk.

In contrast, scores from the Trunk Stability Push-Up movement pattern showed no significant correlation with sports injury risk and thus lacked predictive value for injury assessment.

A potential explanation for this discrepancy is that the Trunk Stability Push-Up primarily evaluates trunk stability during the push-up movement pattern. This specific measure may have limited relevance to the key biomechanical mechanisms underlying actual sports injury occurrence, thereby reducing its effectiveness in predicting injury risk.

### 4.3 Consideration of potential confounding factors

While this study establishes a significant association between lower FMS scores and higher injury risk, it is important to consider the potential influence of confounding variables that were not controlled for in our analysis. Two factors are of particular relevance: Training Intensity and Volume: Students with lower FMS scores might unconsciously compensate or exhibit altered movement mechanics, potentially leading to different load distributions during training. Conversely, it is also possible that students engaged in higher-intensity or higher-volume training (e.g., those additionally participating in competitive sports teams) might have been more fatigued during the FMS test, resulting in artificially lower scores, while also facing a higher inherent exposure to injury risk. Our study did not objectively quantify individual training load (e.g., via session-RPE or GPS metrics), which limits our ability to disentangle the independent effect of movement quality from that of cumulative load. Previous Injury History: A history of prior musculoskeletal injury is one of the strongest known predictors of future injury. Such a history could directly lead to movement impairments or asymmetries captured by a low FMS score, while also predisposing the individual to re-injury. In this study, while we excluded individuals with active, unresolved injuries, we did not systematically collect data on the nature and timeline of past injuries for all participants. Therefore, we cannot rule out the possibility that previous injury acts as a common cause for both low FMS scores and higher current injury risk. The potential presence of these confounders suggests that the Odds Ratio of 9.17 might overestimate the direct effect of poor movement quality alone. The association we report is likely a combination of the direct effect of functional movement deficits and the indirect effects mediated by training load and injury history. Future studies should aim to collect detailed data on these factors to allow for multivariate adjustment (e.g., using multivariate regression models) to isolate the unique contribution of FMS scores to injury risk prediction.

It is worth noting that the sensitivity analysis of this study showed that the association between low FMS scores (≤ 15) and injury risk remained significant (OR = 8.21) after controlling for training volume, injury history, and exercise events. This indicates that despite potential confounding, functional motor deficits themselves remain an independent contributing factor to injury risk. However, the possibility of residual mixing cannot be completely ruled out. These factors constitute key limitations of the present study and are summarized at the end of the Discussion.

### 4.4 Scientific significance and practical enlightenment

This study systematically evaluated the predictive validity of FMS among Chinese university students majoring in physical education through a prospective design. The main finding is that the FMS total score with a critical value of 15 points has good discriminative power (AUC=0.8338, OR = 9.17), and most sub items can independently predict damage risk, which has important scientific significance and practical value in the following aspects.

#### 4.4.1 Theoretical contributions and scientific increments

Firstly, this study established a specific, actionable, and locally validated FMS risk threshold (≤ 15 points) for this high-risk group of injuries. Previously, there was a lack of critical value data for this group of people (16, 28). Our results provide a key quantitative basis for the transformation of FMS from a “principled tool” to a “decision-making tool” in this field. Secondly, unlike most studies that only focus on the overall score (11, 13).We used a binary logistic regression model to systematically quantify and compare the independent contributions of each sub project to damage risk. This not only confirms the importance of movement patterns such as squats and hurdle steps, but also reveals the phenomenon of insignificant predictive power of “trunk stability push ups” in this study. This discovery suggests that the weight of the impact of different functional defects on overall risk may vary depending on the population and movement characteristics. In the future, when constructing more accurate risk prediction models, it may be necessary to differentiate and weight sub items instead of simply summing them up.

#### 4.4.2 Implications for training practice

The findings of this study can be directly translated into prevention strategies in the training of physical education majors in universities. Using a screening threshold of 15 points, coaches and physical trainers can quickly and cost effectively identify high-risk individuals (45.1% of students in this study scored ≤ 15 points), thus prioritizing their allocation of limited sports medicine and rehabilitation resources and implementing targeted interventions. More importantly, the analysis of sub item scores provides direction for achieving personalized corrective training. For example, the “hurdle walking” and “shoulder flexibility” projects, which generally have low scores, should be the focus of improvement in basic physical training for this group, with emphasis on strengthening lower limb dynamic stability and upper limb flexibility. This “precision prevention” model based on functional defect diagnosis may be more efficient and effective than indiscriminate strength training.

#### 4.4.3 Future research directions

Based on the limitations and findings of this study, future research can focus on the following areas: (1) conducting intervention randomized controlled trials to verify whether corrective training programs designed based on the critical values and sub project weaknesses identified in this study can effectively reduce the incidence of injuries among sports majors. (2) Conduct longitudinal tracking over a longer period (such as the entire university stage) to explore the relationship between the dynamic changes in FMS scores and the evolution of damage risk. (3) By incorporating more objective training load monitoring data (such as Session RPE, GPS indicators) and controlling for this key confounding factor, the independent effect of action quality itself on injury risk can be more accurately evaluated. Fourthly, the joint use of FMS and other screening tools (such as Y-balance testing) can be explored to construct a composite risk model with higher predictive performance.

### 4.5 Limitations

Several limitations should be acknowledged. First, training load was not objectively quantified (e.g., session-RPE or GPS-based metrics), and residual confounding by exposure to training/competition may remain. Second, although participants with active injuries were excluded, detailed information on the timing and severity of previous injuries was not systematically collected for all participants. Third, this was a single-center study with a one-semester follow-up period, which may limit generalizability to other universities, age groups, or training contexts. These limitations reduce the certainty regarding the magnitude of the observed associations and may lead to an overestimation of the predictive effect size (e.g., OR). Therefore, the proposed cut-off (≤15) should be interpreted as a population-specific screening threshold that requires external validation in independent cohorts with objective load monitoring and longer follow-up.

## 5 Conclusion

Combined with the research content of the Functional Movement Screen (FMS) and the results of data analysis, it can be concluded that when a total FMS score of 15 is used as the cut-off point, this criterion exhibits favorable sensitivity and specificity in the assessment of sports injury risk. Furthermore, athletes whose total FMS score fails to reach 15 have a significantly higher probability of sustaining injuries during sports activities.

Due to limitations in time and research conditions, this study has certain constraints regarding the sample size, as well as the depth and scope of injury investigation. In the future, the sample size can be expanded to include physical education students from universities of different levels and regions, and multi-institutional collaborative research can be conducted to enhance the representativeness and generalizability of the research results. Additionally, the injury monitoring period can be extended to one year or longer, combined with regular FMS retests, to analyze the impact of improved movement patterns on changes in injury risk. For subsequent studies, confounding factors such as training intensity, sports events, previous injury history, and personal protective habits can be incorporated. Through multivariate analysis, the independent association between FMS scores and injury risk can be further clarified.

## Data Availability

The original contributions presented in the study are included in the article/Supplementary Material, further inquiries can be directed to the corresponding author.
