# Supplementary material for: Injury risk assessment using the functional movement screen in college physical education majors: a prospective cohort study
Source: Front Rehabil Sci. 2026 Mar 26;7:1777826. doi: 10.3389/fresc.2026.1777826 (PMC13062186; doi:10.3389/fresc.2026.1777826)
Supplement: Supplementary file 1 [file Datasheet1.pdf]

TCTR ID : TCTR20251119005

OTHER ID :

Overall Recruitment Status : Completed **(No Results)**

Retrospective registration
This protocol was registered after enrollment of the first participant.

Tracking Information

First Submitted Date : 12 November 2025

First Posted Date : 19 November 2025

Last Update Posted Date : 19 November 2025

Title

Public Title : Feasibility Analysis of Functional Movement Screen in Injury Risk Assessment for Students Majoring in Physical Education in Colleges and Universities

Acronym : No Data

Scientific Title : Feasibility Analysis of Functional Movement Screen (FMS) in Injury Risk Assessment for Students Majoring in Physical Education in Colleges and Universities

Sponsor ID/ IRB ID/ EC ID : Functional Movement Screen

Registration Site : Thai Clinical Trials Registry

URL : https://www.thaiclinicaltrials.org/show/TCTR20251119005

Secondary ID : No Secondary ID

Ethics Review

1. Board Approval : Submitted, approved

Approval Number : 2025

Date of Approval : 18 July 2025

Board Name : Qufu Normal University Biomedical Ethics Approval Document

Board Affiliation : Qufu Normal University

Board Contact : Business Phone : 13869995888 Ext. No Data

Business Email : 13869995888@163.com

Business Address : Qufu Normal University, Qufu City, Shandong Province, China

Sponsor

Source(s) of Monetary or Material Supports : Qufu Normal University

Study Primary Sponsor : Qufu Normal University

Responsible Party : Name/Official Title : shaojie

Organization : Qufu Normal University

Phone : 13869995888 Ext. No Data

Email : 13869995888@163.com

Study Secondary Sponsor : No Study Secondary Sponsor

Protocol Synopsis

Protocol Synopsis : The research objective is to explore the application value of the Functional Movement Screen (FMS) in the sports injury risk assessment of college students majoring in Physical Education (PE), accurately identify students' movement function deficiencies, clarify the corresponding relationship between FMS test data and injury risk, and determine the risk threshold.
For the research design, 71 PE major students who meet the inclusion criteria were selected as subjects. They completed 7 movement tests using the standard FMS kit, with scores recorded, and their sports injury status was monitored through a one-semester follow-up. GraphPad Prism 9.5.0 was used to plot the Receiver Operating Characteristic (ROC) curve for determining the injury risk threshold, while SPSS 27.0 was applied to conduct analyses such as independent samples t-test and binary logistic regression, so as to explore the correlation between the total FMS score, scores of each individual FMS test, and sports injury risk.

URL not available

Health Conditions

Health Condition(s) or Problem(s) Studied : By recruiting 71 students from Physical Education classes, adopting the standard Functional Movement Screen (FMS) test, and combining it with the sports injury records within a semester, a correlation analysis

was conducted.

Keywords : Functional Movement Screen;Sports Injury;Injury Assessment;College and University Students

Eligibility

Inclusion Criteria : (1)Age:greater than 18,less than 20;(2)Educational background:bachelor's degree or above;(3)No history of neuromuscular,cardiovascular,pulmonary,vestibular or rheumatic diseases;(4)No history of cognitive impairment or physical movement disorders;(5)Body Mass Index(BMI):greater than 20,less than 25;(6)No regular medication use

Gender : Both

Age Limit : Minimum : 18 Years Maximum : 20 Years

Exclusion Criteria : (1)Those who have severe adverse reactions during or after training and are not suitable for continuing training;(2)Subjects with poor compliance who fail to follow the experimental process and affect the efficacy evaluation;(3)Cases where subjects voluntarily withdraw;(4)Those who cannot participate in or are unwilling to accept treatment are not included either;(5)Those with acute severe psychological disorders, acute episodes of severe mental illness, or severe impairment of cognitive function

Accept Healthy Volunteers : Yes

Status

Overall Recruitment Status : Completed

Key Trial Dates

Study Start Date (First enrollment) : 26 February 2025	Indicate Type : Actual
Completion Date (Last subject, Last visit) : 08 March 2025	Indicate Type : Actual
Study Completion Date : 08 March 2025	Indicate Type : Actual

Design

Study Type : Observational

Primary Purpose : Screening

Number of Groups : 2

Study Endpoint Classification : N/A

Sample size

Planned sample size : 69

Actual sample size at study completion : 69

Observation Group 1

Group name : Subjects with an FMS score below 15

Group Description : Indicates the presence of motor function deficits, which may be associated with issues such as muscle imbalances and limited joint mobility

Observation Group 2

Group name : Subjects with an FMS score of greater than or equal to5

Group Description : Indicates that the subjects have relatively standardized functional movement patterns, as well as good physical flexibility, stability, and coordination

Outcome

Primary Outcome

1. Outcome Name : diagnosed with injury

Metric / Method of measurement : A score below 15 is defined as being prone to injury

Time point : 6 months after the intervention

Secondary Outcome

1. Outcome Name : Functional Movement Screen

Metric / Method of measurement : Functional Movement Screen (FMS) is a screening method that assesses the quality of basic human movements and identifies muscle imbalances and injury risks

Time point : 6 months after the intervention

Location

Section A : Central Contact

Central Contact First Name : jie Middle Name : Last Name : shao

	Degree :	Phone : 13869995888 Ext. : No Data	Email : 13869995888@163.com
Central Contact Backup	First Name : baixufan	Middle Name :	Lastname : fan
	Degree :	Phone : 15965399861 Ext. : No Data	Email : 13869995888@163.com

Section B Facility Information and Contact

1.	Site Name : Qufu Normal University		
	City : china	State/Province : ShanDong	Postal Code : 273100
	Country : China	Recruitment Status : Completed	
**Facility Contact**	First Name : lei	Middle Name :	Last Name : zhu
	Degree :	Phone : 13562411464 Ext. : No Data	Email : zhulei316@126.com
**Facility Contact Backup**	First Name : shaojie	Middle Name :	Last Name : jie
	Degree :	Phone : 15965399861 Ext. : No Data	Email : 13869995888@163.com
**Investigator Name**	First Name : baixifan	Middle Name :	Last Name : fan
	Degree :	Role : Principal Investigator	

Section C : Contact for Public Queries (Responsible Person)

First Name : jie	Middle Name :	Last Name : shao
Degree : No Data	Phone : 13869995888 Ext. : No Data	Email : 13869995888@163.com
Postal Address : Qufu Normal University, Jining City, Shandong Province		
State/Province : Shandong	Postal Code : 273100	
Country : China	Official Role : Study Principal Investigator	
Organization Affiliation : Qufu Normal University		

Section D : Contact for Scientific Queries (Responsible Person)

First Name : jie	Middle Name :	Last Name : shao
Degree : No Data	Phone : 13869995888 Ext. : No Data	Email : 13869995888@163.com
Postal Address : Qufu Normal University, Jining City, Shandong Province		
State/Province : Shandong	Postal Code : 273100	
Country : China	Official Role : Study Director	
Organization Affiliation : Qufu Normal University		

Summary Results

Date of posting of results summaries : Summary results not yet available

Date of first journal publication of results : Not yet published

Deidentified Individual Participant-level Data Sharing

Plan to share IPD : No

Reason : Concerns over potential identification risks even with anonymization and Participants prior agreement to restrict data use to the original research only.

Publication from this study

MEDLINE Identifier : No Data

URL link to full text publication : No Data

 This document was created on 30 December 2025 at 10:35 (GMT+7).
