# Supplementary figures and images for: Injury risk assessment using the functional movement screen in college physical education majors: a prospective cohort study
Source: Front Rehabil Sci. 2026 Mar 26;7:1777826. doi: 10.3389/fresc.2026.1777826 (PMC13062186; doi:10.3389/fresc.2026.1777826)

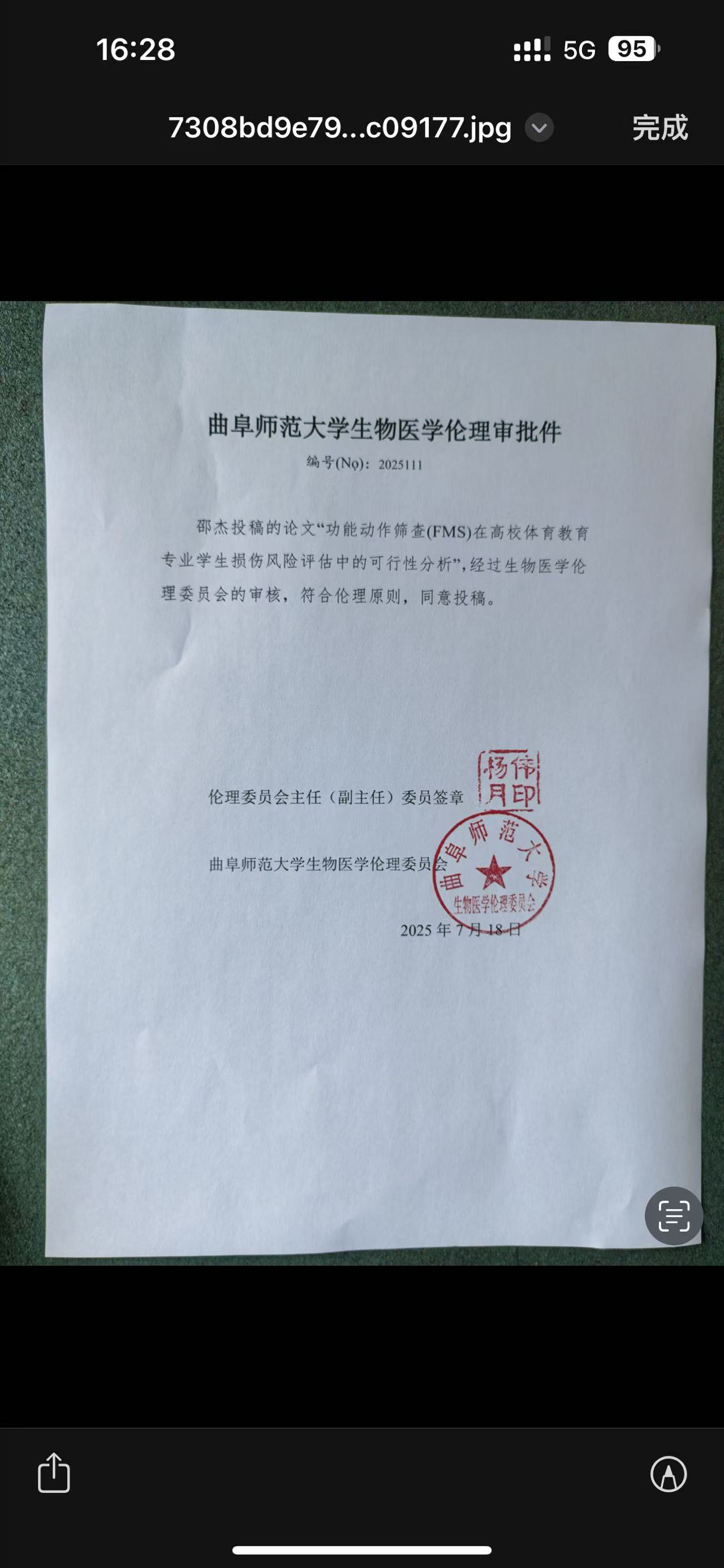

Supplement: Supplementary file 2 [file Image1.jpeg]
